# Personals

**Published:** 1913-08

**Authors:** 


### PERSONALS.

*Announcements of removals, travel, and other matters of interest are requested. Please report any errors in the listing of any physician, in the State and other directories that we may co-operate with the State Society in securing a correct list of physicians.*

---

Dr. Frederick A. Hayes of Buffalo has recently returned from a hunting trip.

---

Dr. F. W. Filsinger of Buffalo announces the removal of his office and residence to 343 E. Utica street.

---

Dr. Wm. G. Telfair of Rochester has associated himself with the new management of the Glenwood Sanitarium of Dansville, N. Y.

Dr. Grover W. Wende of Buffalo is in Europe.

---

Dr. William S. Grimes of Detroit has been appointed Examining Surgeon of the first district of Michigan, including Detroit.

---

Dr. George A. Himmelsbach and Dr. Wm. G. Taylor of Buffalo will attend the Medical Congress in London and proceed to the Continent, returning early in the fall.

---

Dr. H. I. Davenport of Auburn has just returned from a two weeks' automobile tour of Massachusetts, Vermont and eastern New York.

---

Major John B. Coakley, M. D., a Confederate war surgeon, Dr. Arthur W. Hurd and Dr. A. L. Benedict of Buffalo attended the Gettysburg anniversary celebration.

---

Dr. Edith R. Hatch of Buffalo recently spent ten days in Williamsburg, Pa.

---

Dr. A. E. Bartoo has moved from Buffalo to Wilson, N. Y.

---

Dr. Albert E. Woehnert of Buffalo is in Europe.

---

Dr. Ira P. Trevett of Lackawanna attended the Gettysburg anniversary with his father, Mr. L. C. Trevett of the 116th N. Y. Volunteers.

---

Dr. James P. Barr of Buffalo has been fishing in the Muskoka region.

---

Dr. Wm. Brady of Elmira is spending the month of July with his parents in Canandaigua.

---

Dr. C. F. Abbott of Elmira has just returned from a two weeks' trip through New Jersey, Delaware and Maryland.

---

Dr. C. Haase of Elmira has recovered from an attack of diphtheria. In August he sails for Europe to spend four months in Vienna.

Dr. Lee Kinner of Elmira has returned from a two weeks' visit to Chicago.

Dr. Inda of Buffalo, U. B., 1913, has been appointed interne at the Arnot-Ogden Hospital, Elmira, N. Y.

Dr. C. D. Young of Rochester has been away for months in the Thousand Islands and St. Lawrence district.

Dr. James W. Enright, until recently at Pavilion, has removed to Rochester, N. Y., corner of Child and Wilder streets.

Dr. W. E. Powell of Rochester has opened an office at 251 Arnett Boulevard.

Dr. E. H. Wolcott of Rochester is at Montreal and on the St. Lawrence River for a few weeks.

Dr. Edward W. Jackson of Rochester will leave shortly for an extended period of study abroad and then will settle in Rochester permanently.

Dr. E. B. Angell is spending the summer at his cottage on the Lake shore near Rochester.

Dr. D. H. Atwater of Rochester is away on the Jersey coast for several weeks.

Dr. S. W. Little is spending the summer at his cottage on the Lake shore near Rochester.

Dr. M. May Allen of Rochester has been away for a short vacation.

